# Mapping Research on miRNAs in Cancer: A Global Data Analysis and Bibliometric Profiling Analysis

**DOI:** 10.3390/pathophysiology29010007

**Published:** 2022-02-25

**Authors:** Peter Shaw, Kartik Lokhotiya, Chellan Kumarasamy, Krishnan Sunil, Deepa Suresh, Sameep Shetty, Gothandam Kodiveri Muthukaliannan, Siddhartha Baxi, Ravishankar Ram Mani, Palanisamy Sivanandy, Harish C. Chandramoorthy, Madan Mohan Gupta, Suja Samiappan, Rama Jayaraj

**Affiliations:** 1 Oujiang Laboratory, Wenzhou Institute, University of Chinese Academy of Sciences, Wenzhou 325000, China; petershaw@ojlab.ac.cn; 2 Menzies School of Health Research, Darwin 0810, Australia; 3 School of Biosciences and Technology, Vellore Institute of Technology (VIT), Vellore 632014, India; kartik.lakhotiya2016@vitstudent.ac.in (K.L.); gothandam@gmail.com (G.K.M.); 4 School of Health and Medical Sciences, Curtin University, Perth 6102, Australia; chellan.kumarasamy@curtin.edu.au; 5 Department of Radiation Oncology, Mayo Clinic Florida, Jacksonville, FL 32224, USA; krishnan.sunil@mayo.edu; 6 Division of Endocrinology, Department of Internal Medicine, Mayo Clinic Florida, Jacksonville, FL 32224, USA; deepa.suresh@mayo.edu; 7 Department of Oral and Maxillofacial Surgery, Manipal College of Dental Sciences, Mangalore, Manipal Academy of Higher Education, A Constituent of MAHE, Manipal 576104, India; sameep.shetty@manipal.edu; 8 Genesis Care Gold Coast Radiation Oncologist, John Flynn Hospital, Tugun 4224, Australia; siddhartha.baxi@genesiscare.com; 9 Department of Pharmaceutical Biology, Faculty of Pharmaceutical Sciences, UCSI University, Kuala Lumpur 56000, Malaysia; ravishankar@ucsiuniversity.edu.my; 10 Department of Pharmacy Practice, School of Pharmacy, International Medical University, Kuala Lumpur 57000, Malaysia; palanisamySivanandy@imu.edu.my; 11 Stem Cells and Regenerative Medicine Unit, Department of Microbiology and Clinical Parasitology, College of Medicine, King Khalid University, Abha 56000, Saudi Arabia; hshkonda@kku.edu.sa; 12 School of Pharmacy, Faculty of Medical Sciences, The University of the West Indies, St. Augustine 3303, Trinidad and Tobago; madanmohan.gupta@sta.uwi.edu; 13 Department of Biochemistry, Bharathiar University, Coimbatore 641046, India; sujaramalingam08@gmail.com; 14 Northern Territory Institute of Research and Training, Tiwi 0810, Australia

**Keywords:** bibliometric analysis, cancer, miRNA, clinical diagnostics, clinical prognostics

## Abstract

miRNAs biomarkers are emerging as an essential part of clinical oncology. Their oncogenic and tumour suppressor properties playing a role in malignancy has generated interest in their potential for use in disease prognosis. While several studies on miRNA have been carried out across the globe, evaluating the clinical implications of miRNAs in cancer diagnosis and prognosis research has currently not been attempted. A study delineating the area of miRNA research, including the topics presently being focused on, the seminal papers in this field, and the direction of research interest, does not exist. This study aims to conduct a large-scale, global data analysis and bibliometric profiling analysis of studies to evaluate the research output of clinical implications of miRNAs in cancer diagnosis and prognosis listed in the SCOPUS database. A systematic search strategy was followed to identify and extract all relevant studies, subsequently analysed to generate a bibliometric map. SPSS software (version 27) was used to calculate bibliometric indicators or parameters for analysis, such as year and country of affiliation with leading authors, journals, and institutions. It is also used to analyse annual research outputs, including total citations and the number of times it has been cited with productive nations and H-index. The number of global research articles retrieved for miRNA-Cancer research over the study period 2003 to 2019 was 18,636. Between 2012 and 2019, the growth rate of global publications is six times (*n* = 15,959; 90.71 percent articles) that of 2003 to 2011. (2704; 9.29 per cent articles). China published the most publications in the field of miRNA in cancer (*n* = 7782; 41%), while the United States had the most citations (*n* = 327,538; 48%) during the time span. Of these journals, Oncotarget has the highest percentage of article publications. The journal Cancer Research had the most citations (*n* = 41,876), with 6.20 per cent (*n* = 41,876). This study revealed a wide variety of journals in which miRNA-Cancer research are published; these bibliometric parameters exhibit crucial clinical information on performance assessment of research productivity and quality of research output. Therefore, this study provides a helpful reference for clinical oncologists, cancer scientists, policy decision-makers and clinical data researchers.

## 1. Introduction

microRNAs (miRNAs) are small, non-coding RNA that measure 21–23 nucleotides in length and the expression of these miRNAs in the form of miRNA profiles in cancerous states has been found to be highly informative, containing signals for tumour developmental lineage and differentiation state [1]. In comparison to normal tissues, tumours display a general downregulation of miRNAs. Furthermore, researchers have used miRNA expression profiles to identify poorly differentiated tumours, while messenger RNA profiles were ineffective when applied to the same samples. These findings demonstrate the utility of miRNA profiling in research [1].

Since their discovery in 1993, microRNAs (miRNAs) have been proven to play critical roles in gene regulation [2,3]. MiRNAs were previously assumed to be degraded RNA fragments, but their importance in cancer research and therapy became clear with the discovery of their aberrant expression in a variety of clinical events and their role in carcinogenesis [3,4,5,6,7,8,9,10]. The most important characteristic of miRNA is its stability and longevity while being stored [3,11,12,13,14,15,16,17]. They are well preserved in extracted blood, formalin-fixed tissues, and paraffin-embedded tissues [3,18]. This allows scientists to run a variety of analyses on a large number of tissue samples, presenting them with massive amounts of data.

MiRNAs are hypothesised to have a dual function in carcinogenesis, acting as both oncomirs and tumour suppressors [19,20,21]. This theory is reinforced by the fact that miRNA expression in tumours can be up- or down regulated in contrast to normal tissue [3,22,23,24,25,26,27,28,29,30,31,32,33,34]. Tumour miRNA expression analysis has provided a lot of conceptual understanding of tumour differentiation and lineages [3,35,36,37,38,39]. MiRNA signatures can be used as a diagnostic tool, prognosis variable, predictive factor, prospective therapeutic target, and pharmacodynamics marker, in addition to providing sufficient information on tumour biology [3,40,41,42]. Multiple studies have assessed the prognostic utility of miRNA, with few studies highlighting specific miRNA’s that have a significant prognostic effect across multiple patient populations [43,44,45,46,47,48,49,50,51,52,53].

Circulating miRNAs have the potential to be employed as biomarkers in diagnostic, prognostic, and therapeutic studies [46,54,55,56,57,58,59,60,61,62]. Although the stability of miRNAs assists in determining their presence in the blood, the majority of procedures are performed using biopsies collected from primary tumours and metastases [63,64]. Without attempting to provide a thorough overview, we have illustrated the clinical feasibility of miRNAs being utilised as biomarkers from any of the aforementioned sources [1,3,65]. Accurate miRNA studies can be used to monitor carcinogenesis and its progression [66,67,68]. A precise and personalised approach provides a higher success rate and clinical efficacy than traditional medicines [69,70].

## 2. Study Objective

The objective of this study was to examine a large-scale, global data analysis and bibliometric profiling analysis of studies to provide an in-depth evaluation of the research output of miRNAs in cancer diagnosis and prognosis listed in the Scopus database. Statistics and data extracted from Scopus ascertain the diagnostic and prognostic role of miRNA in cancer and its malignancy. In the era of precision oncology, determining the aberrant expression of miRNA and its role in cancer development and progression can help better understand tumour biology and aim at personalised and targeted therapies. A detailed study of the field of miRNA and cancer would help researchers learn more about current scientific patterns and to gain insight into the contributions made by particular countries, institutions, partnerships, and contributing authors. The h-index could be used to rank universities, apply for grants, and create a scientific reputation in the scientific community.

## 3. Methods

The data for this study was extracted from the Scopus database. Scopus is an Elsevier database that includes comprehensive Medline coverage for over 20,000 publications and has a 20% coverage edge over Web of Science. The following Scopus attributes were used as data items for this study: citation analysis, contribution by nation and author, source names, and productivity per year. For the search of studies, the investigation period was set between 2003 and 2019. Search engines such as Scopus was used to select subject categories consisting of life, social, health, and physical sciences.

All documents featuring “miRNA in cancer” as the primary keyword were screened. Further refinement of the search was carried out using the categories of “country”, “author”, “affiliation”, “source” and “year”. Subsequently, using Scopus’ citation tracker, all relevant papers published in Scopus were retrieved and examined. The following inclusion/exclusion criteria were used for the secondary screening of the full-texts of studies.

### 3.1. Selection Criteria

- The keywords were chosen based on the number of queries and the bibliometric graph’s density variation.
- These keywords appear in the majority of miRNA in cancer research papers.

### 3.2. Inclusion Criteria

- miRNA expression papers focused on cancer prognosis or diagnosis or clinical cancer or clinical outcomes or treatments.
- The definition also comprises studies that are designed to evaluate the miRNAs that are downregulated or upregulated in cancer patients (specifically the magnitude of down/upregulation and their impact on patient prognosis).
- Manuscripts in all languages were included.
- All papers published in Scopus journals were included in the study to improve the precision of our search.
- All records, including errata, articles, book chapters, and conference papers, were included, making this a systematic review that included all sources related to miRNA in cancer and covered a broad range of research.
- Documents with clinical prognosis in cancer patients and patients’ survival or recurrence related to clinical diagnoses were considered.

### 3.3. Exclusion Criteria

Manuscripts that were labelled as abstracts, dissertation reports, case reports, case series, or un-defined types of documents were excluded.

The collected data were transferred to the MS Office Excel sheet that permitted us to collate the information contained in the records and to retrieve bibliometric indicators. After refining the findings with Scopus software online, all the documentation and results were collected. All the values retrieved from the Scopus website were collected and analysed after sorting by keywords.

### 3.4. Global Data and Bibliometric Profiling Analysis of Studies

The validity of our strategy was tested by observing the first 100 most often cited documents retrieved by utilising the procedure mentioned above. All the documents were published in high impact, reputed oncology journals.

The important indicators for analysis offered in this study were

- The form and language of published documents,
- Nation and institutional affiliation,
- Source/journal-title in which documents were published,
- Most active authors,
- Most cited papers,
- Collaboration trends and

Their related h Index. (Difference in the number of articles published during that period/number of articles published at the start of the period) × 100 was used to calculate the growth rate of production.

### 3.5. Publication Productivity

The leading authors and institutions of miRNA-Cancer research in this field.

### 3.6. Overview of the Research Output and Growth of miRNA-CANCER Research

A number of research indicators were included in order of importance. The total number of references was used to identify the most important papers in the subject, while the number of publications was used to estimate research production.

### 3.7. Core Bibliometric Indicators

The Scientific Journal Ranking (SJR) of journals were obtained using the SCImago Journal and Country Ranking website as a measure of journal quality.

The Hirsch index (h-index) was used to determine the number and quality of publications by region, institution, and author. When a country or an entity publishes x articles, each of which has at least x citations., it earns an h-index of x.

The World Bank’s online databases (Countries and Economies, http://data.worldbank.org/nation, accessed on 1 July 2020) is used to standardise the research effectiveness of different countries are ranked by population and national gross domestic output (GDP).

Other parameters for analysis were estimated using SPSS software. An excel graph analyses the year, nation, number of citations, number of publications, GDP, population, h-index, affiliation, and source. All calculations, such as average and total, were performed after creating appropriate tables from the data obtained from Scopus.

The IRB ethics approval for the investigation was exempted as the information was obtained from publicly available electronic sources and did not relate to specific patients’ information or profiles.

## 4. Results

### 4.1. General Data

**Figure 1:**
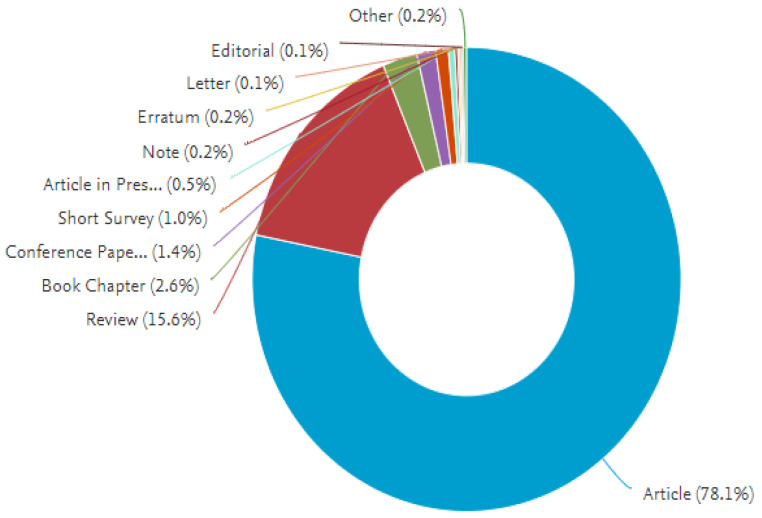
Document types of retrieved articles on miRNA in cancer (2003–2019).

**Figure 2:**
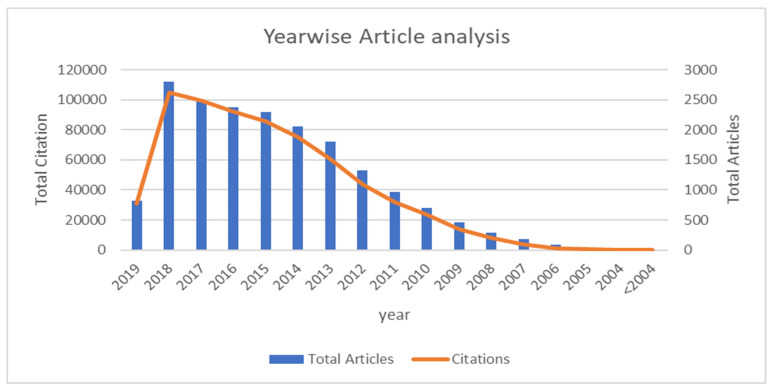
Year-wise article analysis.

Between 2003 and 2019, a total of 18,663 journal papers were retrieved. About 70.1 percent of the submissions were research articles, while the remaining 21.9 percent were review posts, book chapters, conference papers, short surveys, articles in the press, notices, erratum, letters, editorials, books, retracted articles, and conference reviews (Table 1). Figure 1 depicts the document type distribution as a pie map. English was the most frequently encountered language in the article retrieval (*n* = 18,067; 96.81 per cent), with Chinese, Japanese, Russian, German, Czech, French, Spanish, Polish, and Persian accounting for the remainder (*n* = 588, 3.19 per cent) (Table 2) (Figure 2).

### 4.2. Publications with Time

When compared to the first half of the time period between 2003 and 2011 (2704; 9.29 per cent articles), the number of retrieved published papers increased by about six times (*n* = 15,959; 90.71 percent articles) in the second half of the time period from 2012 to 2019. Between the first half of the study period (2003–2011) and the second half (2012–2019), the number of citations increased sevenfold. In 2017, the overall number of citations per paper published was the highest, but in 2018, the number of publications and citations published was the highest. For the entire time span, the average citation per document was 36.19. Figure 2 shows the frequency of publication and citation over the period of pre-2004 to 2019.

Table 3 displays the total number of articles retrieved, together with their access type and the number of citations per year, as well as the average number of citations per document.

### 4.3. Countries

**Figure 3:**
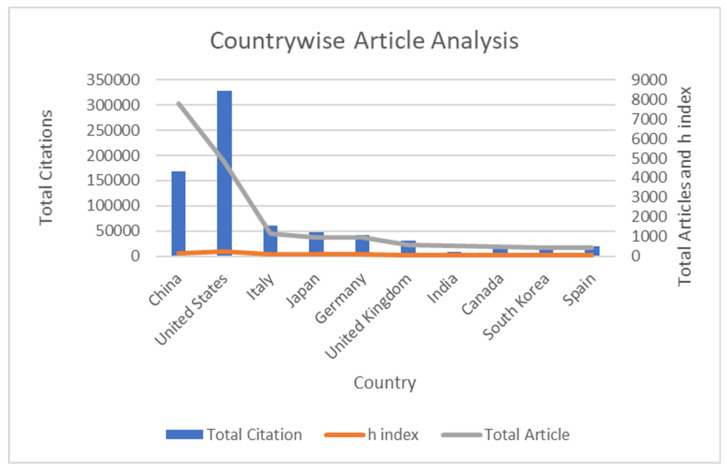
Country-wise article analysis.

**Figure 4:**
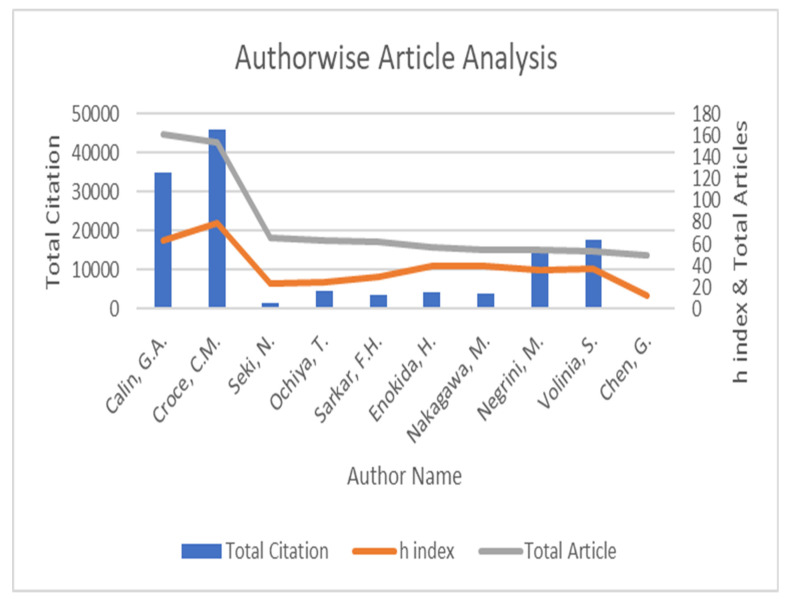
Author wise article analysis.

There were 106 countries represented in the publications on miRNA in cancer, as well as 150 papers from unidentified places (Figure 3). Table 4 lists the top ten countries by a number of papers published and citations, as well as their h-indices. China published the most publications in the field of miRNA in cancer (*n* = 7782; 41%), while the United States had the most citations (*n* = 327,538; 48%) during the time span. China and the United States contributed approximately 68 per cent of article publishing, while Italy, Japan, Germany, the United Kingdom, India, Canada, South Korea, and Spain contributed approximately 29 per cent. Figure 4 shows the country-wise frequency of publication and citation, on the topic of miRNA mapping and cancer.

### 4.4. Most Frequent Terms

#### 4.4.1. Authors

Professor G.A. Calin came in first with the most publications (161) and citations (h-index) of all of the researchers. With 46,117 citations and an h-index of 79, Professor Croce, C.M., came in first. Both came from the United States. Table 5 lists the top ten writers in terms of the number of publications. Figure 4 shows the total number of citations and articles by writers, as well as their h-indices and C/A values.

#### 4.4.2. Frequently Cited Articles

“MicroRNA expression profiles identify human cancers”, by Lu et al., was published in Nature in 2005 and received the most citations. As of the time of data collection, the article had 6712 citations. Table 6 lists the top ten most cited papers on miRNA in cancer. Eight of the ten documents were original papers, with only two being reviews.

#### 4.4.3. Journals

Table 7 lists the top ten journals that published papers about miRNA in cancer from 2003 to 2019. These ten publications accounted for roughly 20% of all papers retrieved. With 4.44 per cent (*n* = 829) of the total retrieved publications, the journal Oncotarget has the highest percentage of Article publications. The journal Cancer Research had the most citations (*n* = 41,876), with 6.20 per cent (*n* = 41,876). It had the highest h-index of 106 as well. Figure 4 depicts a source-by-source review of papers and citations, as well as the h-index. Seven of the top ten publications are dedicated to cancer and tumour biology.

#### 4.4.4. Subject Area

Biochemistry, Genetics, and Molecular Biology (*n* = 12,906; 42.2 percent) had the most publications (*n* = 10,193; 33.3 percent), led by Medicine (*n* = 10,193; 33.3 percent). Pharmacology, Toxicology, and Pharmaceutics (*n* = 1510; 4.9%), Agricultural and Biological Sciences (*n* = 1045; 3.4%), Chemistry (*n* = 780; 2.5%), Immunology and Microbiology (*n* = 777; 2.5%), Computer Science (*n* = 527; 1.7%), Chemical Engineering (*n* = 476; 1.6%), Engineering (*n* = 448; 1.5%), Neuroscience (*n* = 414; 1.4%), Others (*n* = 1527; 5.0%).

Funding sponsor Table 8 shows the top 10 funding agencies for articles on miRNA in cancer.

## 5. Discussion

### 5.1. Key Findings

Bibliometric assessment was used to illustrate various aspects of global scientific evidence on miRNA and its vital role in cancer, as well as the ongoing development of publications in this area from 2003 to 2019. Scopus was used to examine the research papers published in the title related to miRNA and its function in cancer research. A total of 14,572 articles were reported and published between 2003 and 2019. From 2012 until 2019, the second half of the time period, the number of retrieved research papers expanded by about six times as compared to the first half of the time period from 2003–2011. China and the United States accounted for almost two-thirds (68%) of all written papers, while Italy, Japan, Germany, the United Kingdom, India, Canada, South Korea, and Spain each contributed about a fifth (29%).

### 5.2. Journals That Published the Most Articles

According to our findings, the journal “Oncotarget” was the most productive in terms of publishing papers about miRNA in cancer, whereas Plos One, Oncology Reports, Oncology Letters, Tumor Biology, Molecular Medicine Reports, Cancer Research, Scientific Reports, Oncogene, and International Journal of Oncology were actively involved in publishing work related to miRNA in cancer. The journal “Cancer Research” had the highest number of citations and h-index when compared to the other nine journals mentioned above.

### 5.3. Good Quality Publication Output

The rising number of high-quality studies validates the role of miRNA in cancer. Using bibliographic and density visualising analysis of publications indexed in Scopus, the global research output on research that investigates the relationship between miRNA and cancer was calculated using three indices of qualitative research outputs: total citations, h index, and average citation (45). These vital bibliometric indices were calculated for the top ten productive authors, journals, countries, and institutions in this present study. Professor Croce, C.M., from the United States, has total citation numbers of 46,117 and an h-index of 79, whereas Lu et al., from the United States, had received the highest number of citations for the article entitled “MicroRNA expression profiles classify human cancers”.

### 5.4. Strengths

The study is novel for its comprehensive extraction and evaluation of research outputs from the available global data and for establishing the clinical ramifications of miRNAs in cancer diagnosis and prognosis.

### 5.5. Limitations

The limitation of our exploratory search is the exclusion of publications from non-Scopus bibliographic sources like PubMed, Google Scholar, and Web of Science. The extraction of data from Scopus encompasses vital bibliometric and visual density processing indicators and can provide a credible and precise impression of the output of publications.

In addition, the global data analysis of total citations and citations per article may be confounded with self-citations by authors. Furthermore, this bibliometric study did not collect distinct institutional affiliations as well as single and multiple country publications as specific information was not available for all articles, and miRNA-Cancer research is primarily led by research groups affiliated with one institution.

## 6. Conclusions

This study was a comprehensive bibliometric analysis of studies contemplating the role of miRNAs in cancer. Scopus database was exclusively used to collect papers relevant to the study, articles from various journals in Scopus were scanned, and relevant data were retrieved. During the time period from 2003 to 2019, it was noted that the number of papers published in the time span of the years 2012 to 2019 had increased by six times in contrast to the number of papers published from 2003 to 2011, while the number of citations increased sevenfold in the same pattern.

The bibliometric indicators validate the vital clinical information on performance assessment of research productivity and quality of research output. Hence, this study ascertains a valuable reference for clinical oncologists, cancer scientists, policy decision-makers and clinical data researchers.

## Figures and Tables

**Table 1:** The number and frequency of different source types based on bibliometric search for the topic of miRNA research in cancer.

Document Type	Number	Frequency %
Article	14,572	78.08%
Review	2909	15.59%
Book Chapter	485	2.60%
Conference Paper	266	1.43%
Short Survey	178	0.95%
Article in Press	89	0.48%
Note	40	0.21%
Erratum	31	0.17%
Letter	26	0.14%
Editorial	25	0.13%
Book	17	0.09%
Retracted	15	0.08%
Conference Review	10	0.05%
Total	18,663	100.00%

**Table 2:** Top 10 languages of retrieved articles on miRNA in cancer (2003–2019).

	Language	Total No. of Articles	% of Articles
1st	English	18,067	96.81%
2nd	Chinese	463	2.48%
3rd	Japanese	24	0.13%
4th	Russian	22	0.12%
5th	German	20	0.11%
6th	Czech	15	0.08%
7th	French	15	0.08%
8th	Spanish	13	0.07%
9th	Polish	9	0.05%
10th	Persian	7	0.04%

**Table 3:** Number of published articles and citations per year (2003–2019).

Year	Open Access	Closed Access	Total Articles	Citations	Average Citation per Document
2019	296	520	816	30,808	37.75490196
2018	1324	1486	2810	104,985	37.36120996
2017	1344	1132	2476	99,818	40.31421648
2016	1199	1175	2374	91,943	38.72914912
2015	1204	1091	2295	86,001	37.47320261
2014	1037	1026	2063	75,003	36.35627727
2013	882	920	1802	60,639	33.6509434
2012	672	651	1323	43,723	33.04837491
2011	438	533	971	32,016	32.97219361
2010	314	380	694	23,399	33.71613833
2009	216	238	454	13,884	30.5814978
2008	146	147	293	8028	27.39931741
2007	90	87	177	3607	20.37853107
2006	41	41	82	1249	15.23170732
2005	11	15	26	326	12.53846154
2004	2	2	4	51	12.75
<2004	1	2	3	11	3.666666667
Total (<2004–2019)	9217	9446	18,663	675,491	483.9227894
Average (<2004–2019)	542.1764706	555.6470588	1097.823529	39,734.76	53.76919883

**Table 4:** Top 10 productive countries in miRNA in cancer article publication.

Country Name	Total Citation	h Index	Total Article	% Article	% Citation
China	168,314	145	7782	0.416974763	0.249172824
United States	327,538	244	4832	0.258908	0.48488877
Italy	61,473	108	1137	0.060922681	0.091004913
Japan	47,847	106	958	0.051331512	0.07083292
Germany	42,641	102	946	0.050688528	0.063125934
United Kingdom	30,325	76	567	0.030380968	0.04489327
India	8205	47	515	0.027594706	0.01214672
Canada	19,212	73	496	0.026576649	0.028441534
South Korea	14,240	59	459	0.024594117	0.021080962
Spain	19,244	66	422	0.022611584	0.028488907

**Table 5:** Top 10 authors publishing on miRNA in Cancer.

Author Name	Total Citation	h Index	Total Article	% Article	% Citation	C/A
Calin, G.A.	34,959	63	161	0.008627	0.05175347	217.136646
Croce, C.M.	46,117	79	154	0.008252	0.06827182	299.461039
Seki, N.	1597	24	65	0.003483	0.00236421	24.5692308
Ochiya, T.	4655	25	63	0.003376	0.00689128	73.8888889
Sarkar, F.H.	3723	30	62	0.003322	0.00551155	60.0483871
Enokida, H.	4110	40	57	0.003054	0.00608446	72.1052632
Nakagawa, M.	3953	39	55	0.002947	0.00585204	71.8727273
Negrini, M.	15,135	36	54	0.002893	0.02240592	280.277778
Volinia, S.	17,556	37	53	0.00284	0.02598998	331.245283
Chen, G.	497	12	49	0.002626	0.00073576	10.1428571

**Table 6:** Top 10 cited articles on miRNA in cancer (2003–2019).

Authors	Title	Year	Source Title	Document Type	Cited by
Lu, J. et al. [1]	MicroRNA expression profiles classify human cancers	2005	Nature	Article	6712
Calin, G.A. and Croce, C.M. [4]	MicroRNA signatures in human cancers	2006	Nature Reviews Cancer	Review	5205
Esquela-Kerscher, A. and Slack, F.J. [20]	Oncomirs—MicroRNAs with a role in cancer	2006	Nature Reviews Cancer	Review	5058
Mitchell, P.S. et al. [15]	Circulating microRNAs as stable blood-based markers for cancer detection	2008	Proceedings of the National Academy of Sciences of the United States of America	Article	4606
Volinia, S. et al. [71]	A microRNA expression signature of human solid tumours defines cancer gene targets	2006	Proceedings of the National Academy of Sciences of the United States of America	Article	4193
Iorio, M.V. et al. [72]	MicroRNA gene expression deregulation in human breast cancer	2005	Cancer Research	Article	2928
Johnson, S.M. et al. [73]	RAS is regulated by the let-7 microRNA family	2005	Cell	Article	2729
Landgraf, P. et al. [74]	A Mammalian microRNA Expression Atlas Based on Small RNA Library Sequencing	2007	Cell	Article	2417
Yanaihara, N. et al. [75]	Unique microRNA molecular profiles in lung cancer diagnosis and prognosis	2006	Cancer Cell	Article	2329
Skog, J. et al. [76]	Glioblastoma microvesicles transport RNA and proteins that promote tumour growth and provide diagnostic biomarkers	2008	Nature Cell Biology	Article	2298

**Table 7:** Top 10 journals.

Journal Name	Total Citation	h Index	Total Articles	% Citation	% Articles
Oncotarget	15,560	54	829	2.30%	4.44%
Plos One	33,313	86	777	4.93%	4.16%
Oncology Reports	5462	37	326	0.81%	1.75%
Oncology Letters	2783	26	324	0.41%	1.74%
Tumour Biology	5916	17	303	0.88%	1.62%
Molecular Medicine Reports	2454	23	291	0.36%	1.56%
Cancer Research	41,876	106	280	6.20%	1.50%
Scientific Reports	3945	32	265	0.58%	1.42%
Oncogene	21,939	84	213	3.25%	1.14%
International Journal Of Oncology	5918	44	203	0.88%	1.09%

**Table 8:** Top 10 funding sponsors for articles on miRNA in cancer (2003–2019).

Rank	Founding Sponsor	Number
1	National Natural Science Foundation of China	1440
2	National Natural Science Foundation of China (NSFC)	1205
3	National Institutes of Health (NIH)	521
4	National Institutes of Health	433
5	National Cancer Institute	235
6	National Cancer Institute (NCI)	169
7	National Research Foundation of Korea (NRF)	160
8	National Basic Research Program of China (973 Program)	149
9	Natural Science Foundation of Jiangsu Province	146
10	Associazione Italiana per la Ricerca sul Cancro	122

## Data Availability

Not Applicable.
